# Effects of Group Counseling on Stress and Gender-Role Attitudes in Infertile Women: A Clinical Trial

**Published:** 2019

**Authors:** Zeinab Ehsan, Mansooreh Yazdkhasti, Mitra Rahimzadeh, Mina Ataee, Sara Esmaelzadeh-Saeieh

**Affiliations:** 1- Student Research Committee, School of Medical Sciences, Alborz University of Medical Sciences, Karaj, Iran; 2- Non-communicable Diseases Research Center, Alborz University of Medical Sciences, Karaj, Iran; 3- Social Determinants of Health Research Center, Alborz University of Medical Sciences, Karaj, Iran; 4- Department of Obstetrics and Gynecology, Clinical Research Development Center of Kamali Hospital, School of Medical Sciences, Alborz University of Medical Sciences, Karaj, Iran

**Keywords:** Counseling, Gender role, Infertility, Stress

## Abstract

**Background:**

Infertility stress can have a devastating impact on the lives of couples and influence their physical and psychological health. The purpose of this study was to investigate the effects of group counseling on female stress and gender-role attitudes in infertile women.

**Methods:**

The present study is a randomized clinical trial conducted on 90 infertile women referred to Rooyesh Infertility Treatment Center in the city of Karaj, Iran. The convenience sampling method was used. Samples were divided into intervention and control groups through four-block random allocations. Accordingly, the intervention group received five-session group counselling and the control group only received routine care. Newton’s fertility problem inventory (FPI) and gender role questionnaire (GRQ) were used for collecting data before, after, and one month after the intervention. The significance level was set at 0.05.

**Results:**

The result showed a significant relationship between gender role attitude and stress in infertile women (p=0.03) and indirect association between of them (r=0.13). And also repeated measures test indicated that length of time had affected the total scores of infertility stress (p<0.001) and gender role attitude scores (p= 0.001) and there was a significant difference between the two groups in infertility stress scores (p<0.001) and gender role attitude scores (p=0.001).

**Conclusion:**

Group counseling can be used in stress reduction and also improved gender role attitude of infertile women.

## Introduction

Infertility is defined as failure to achieve pregnancy after one year of continuous normal sexual intercourse without the use of contraceptive methods (1). By this definition, approximately 15% of couples in the world are infertile (2). The prevalence rate of infertility has also been reported to be 20.2% in Iran (3, 4). Male factor was responsible for 34.0%, female factor for 43.5%, both factors for 17.1% and 8.1% were unexplained (5).

Diagnosis of infertility is accompanied by physical and psychological stress for couples (6). However, women suffer from higher levels of stress and tensions than men (7). Assisted reproductive technology (ART) is costly and sometimes contains several cycles that can be economically, emotionally, and physically stressful especially for women. ART is not even standardized or guaranteed, and the most frequent stress is related to making decisions to discontinue infertility treatments and consider a childless life (8, 9).

Infertile women may experience double loss–the loss of opportunity for motherhood and loss of meaningfulness, which both cause social isolation and high levels of stress in a person (10). Numerous factors can predict stress in infertile women including gender-role attitude which is related to views and opinions by individuals about their gender-related behaviors (11).

Gender-role attitudes refer to one’s views about appropriate characteristic behaviors for a given gender (12). Childbirth has been always considered as one of the most prominent characteristics of female role since the past years. Hence, fertility has been considered as a female issue (13). Relationship between gender-role attitude and infertility stress has been reported in numerous studies highlighting the fact that women with traditional attitudes towards female roles were more stressed out (14).

Result of a qualitative study in Iran showed that most of infertile women had experienced stress and uncertainty because they did not know what will happen to them at the end of their efforts. And also they had suffered the burden of treatment only because of their husbands. These women were very concerned about their husbands’ dissatisfaction (15).

Considering that the chance of pregnancy is expected to be multiplied following continued treatments, it seems that providing a strategy to reduce stress in infertile women is appropriate and significant (16). Some studies have illustrated the effectiveness of psychological interventions and cognitive therapies in stress reduction in infertile couples (17–19).

Group counselling in infertile women can impact on sharing of experiences, strengthening communication skills, better understanding of relaxation techniques and psychological support (20, 21).

A study in Iran showed that emotional and psychosocial aspects of treatment should not be ignored as they are important factors for increasing the quality of treatment. Clinicians do not have enough time to talk with patients. Therefore, these problems should be solved through extra counselling program (22).

In this study, an attempt was made to design a counselling program with regard to Iranian culture and gender-role attitudes for reducing stress in infertile women.

## Methods

This research was a randomized clinical trial. Researcher was a graduate student in Midwifery Counseling. She had also received training concerning the content of the research sessions. After obtaining permission from relevant authorities, the researcher referred to Rooyesh infertility center daily. This center at the time of study was the only infertility center in Alborz province and was established 4 years ago. Researcher explained the goals of the research and received oral and written consent from participants. Sampling method in this study was convenient and lasted from February 2018 until May 2018. For group allocation, the four block randomization method was used. There were six possibilities to randomly allocate the samples to the blocks (BABA, BBAA, ABBA, AABB, ABAB, and BAAB). For this purpose, at the onset of the study, one of the blocks was selected and four participants were placed in that block, so block A was selected as intervention group and block B was assigned to the control group. Place for counselling was located at conference meeting room of Kamali Hospital which is the most common referral hospitals in Alborz Province away from Rooyesh Infertility Treatment Center for preventing contact of participants in intervention and control group.

The groups were determined and study’s questionnaires had been completed before, and 1 month after intervention in each group. Control group completed questionnaire in Rooyesh Center and intervention group before and after intervention completed questionnaires in Kamali Hospital and 1 month after intervention completed questionnaires in Rooyesh Center. Before completion of questionnaires in Rooyesh Infertility Center, participants were contacted via phone and appropriate time was determined.

### Participant characteristics:

Participants of study were infertile women referred to Rooyesh Infertility Treatment Center. According to Rabiepour’s study and considering 95% confidence interval and 90% test power, and also %10 sampling drop, sample size was estimated by 45 individuals in each group (23). The inclusion criteria were medical diagnosis of infertility, primary infertility, Iranian nationality, and female infertility, fluency in Persian, minimum reading and writing skills, as well as age range of 22–44 years. The exclusion criteria in this study contained getting pregnant during the infertility treatment and study’s time, having self-reported psychiatric problems, being absent in at least two counseling sessions and having a history of *in vitro* fertilization (IVF) failure.

**Figure 1.**
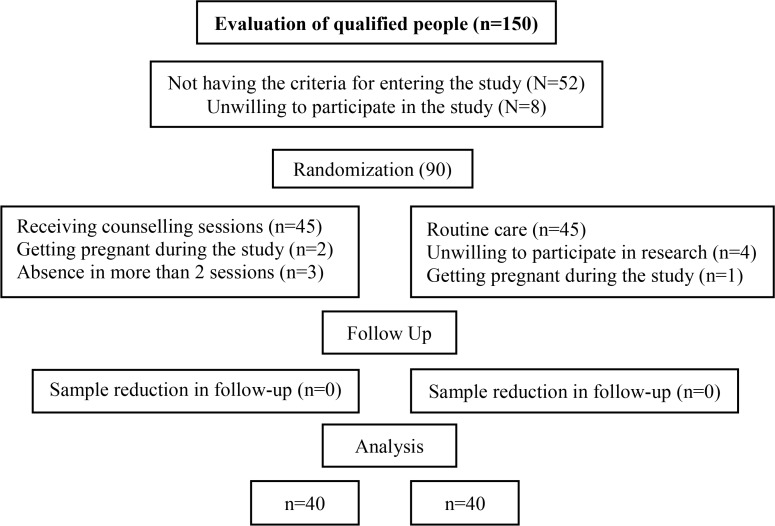
Consort diagram

In the intervention group, 3 individuals were excluded from the study due to lack of participation in more than two counseling program sessions and 2 participants were also excluded for their pregnancy. In the control group, 4 individuals were excluded from the study because of their unwillingness to participate in the research and one person due to her pregnancy. Finally, the study continued with 40 individuals in the control group and 40 individuals in the intervention group (Figure 1). Research analysis was based on pre protocol analysis.

### Intervention:

The infertile women placed in the intervention group received five group counselling sessions twice a week for 90 minutes via lecture, question and answer, and role-play according to the guideline by the British Infertility Counselling Association (BICA), gender-role attitudes, reproductive health and rights, and stress reduction techniques. The guideline by the BICA was first published in 2006 and the third and last version of this protocol was developed and released in 2012 as a frontrunner in the development of a guideline at national and international levels (24). The counselling groups were five groups consisted of nine individuals. In addition to group counselling, the content of counseling sessions was provided through social networking channels and participants’ questions were also answered via phone calls.

In this study, the control group received only routine care (familiarity with causes of infertility and treatment methods). At the end, a handbook with the content of the counseling program sessions was given to the control group to observe ethical consideration in the research.

The content of counselling sessions was approved by the faculty members of the Midwifery Department, Obstetrics and Gynecology Department, and Department of Psychiatry at Alborz University of Medical Sciences as follows:

*Session 1:* Illustration of research objectives along with providing information about infertility, emotional outcomes of infertility, reasons behind emotional outcomes and their relationship with infertility, therapeutic protocols and methods, drug use, as well as pharmaceutical complications and their success rates,
*Session 2:* Role of stress on fertility treatments, identification of women’s perceptions and feelings about infertility in group discussions and listening to their views and opinions, stress reduction techniques, relaxation training (1. mind-muscle technique and 2. muscle-mind technique), relaxation training with mental imagery, focus on breathing techniques, and homework assignment for practicing relaxation techniques on a daily basis until the next counseling session,
*Session 3:* Talks about marital relationships, sexuality, explanations about sexual cycle and impact of infertility on it, sexual dysfunction and ART, problem-solving strategies, communication skills with husband and others, adaptation to conditions, talks about women’s experiences in social and marital relationships, practice and repetition of stress reduction techniques, relaxation training, and homework assignment for practicing relaxation techniques on a daily basis until the next counseling session,
*Session 4:* Defining gender-role attitude and its types, women’s sexual and reproductive rights, discussions about general, sexual, and marital roles of women; women’ childbearing role and its development in recent years, impact of children on couples’ lives, participants’ experiences with absence of children and role of children in family, practice and repetition of stress reduction techniques, relaxation training, and daily homework assignment for practicing relaxation techniques,
*Session 5:* Practicing and repeating stress reduction and relaxation techniques and recommending daily relaxation ones, and suggesting exercises especially walking as well as having a diet for stress reduction.

Data collection questionnaires in this research also consisted of three section including 1. demographic characteristics, 2. Newton’s fertility problem inventory (FPI), and gender role questionnaire (GRQ).

### Newton’s FPI:

This 46-item questionnaire was developed by Newton in 1999 to diagnose infertility stress within five domains. The total score of questionnaire is between 46 and 276 in which higher numerical values could represent higher levels of stress (25). The Persian version of the FPI was psychometry by Samani et al. (26).

### Gender role attitude questionnaire:

This 33-item questionnaire was designed and validated by Humami et al. (9) to measure gender-role attitude in family in two domains (Marital general roles: 0–95 and Marital roles in sexual and reproductive issues: 0–70) of general marital roles and marital roles in sexual and reproductive issues. Responses were made on a 6-point Likert scale and categorized.

### Data analysis:

In this research, the data were analyzed using SPSS Software (Version 16.0). Normality of the data was also investigated via Kolmogorov-Smirnov test. To describe quantitative variables, the mean and standard deviation were reported. T-test was used to compare quantitative variables between the two study groups. Chi-square test and Fisher’s exact test was used to compare qualitative variables. To compare the mean scores of infertility stress and gender-role attitudes between both study groups before, after, and one month after the intervention, repeated measures analysis of variance (ANOVA) was used. The p-value less than 0.05 was considered significant. It should be noted that data analysis was carried out based on pre-protocol analysis.

### Ethical considerations:

This study was approved by the Vice-Chancellor’s Office for Research at Alborz University of Medical Sciences with the code number of Abzums.Rec.1396.161 and also recorded in Iranian Registry of Clinical Trials code number: IRCT20150119020719N6. Before the study, its objectives and the confidentiality of all information were explained to the participants. Informed consent was also obtained from all the participants and the researcher tried to observe all the material and spiritual rights of the study samples.

## Results

In this study, women had a mean age of 30.8± 6.2; all women were married for 6.6±3.7 years, and were attempting to have a child for an average of 6.1±4.2 years. There were no statistically significant differences between two groups. Characteristics of participant are mentioned in table 1.

**Table 1.** Demographic characteristics of infertile women

**Variable**	**Intervention**	**Control**	**p-value**

	**Frequency (%)**	**Frequency (%)**	
**Age (year)**			
22–25	7(17.5)	9(22.5)	0.901 ^*^
26–30	12(30)	9(22.5)	
31–35	10(25)	12(30)	
36–40	8(20)	8(20)	
41–44	3(7.5)	2(5)	
Total	40(100)	40(100)	
Mean±SD	30.83±6.6	30.80±5.8	
**Education**			
Primary school only	2(5)	0	0.162 ^***^
High school degree	21(52.5)	16(40)	
University degree	17(42.5)	24(60)	
Total	40(100)	40(100)	
**Job**			
Homework	32(80)	31(77.5)	0.785 ^**^
Practitioner	8(20)	9(22.5)	
Total	40(100)	40(100)	
**The duration of infertility (year)**			
1–5	23(57.5)	21(52.5)	0.921 ^**^
6–10	11(27.5)	11(27.5)	
11–15	4(10)	6(15)	
16–20	2(5)	2(5)	
Total	40(100)	40(100)	
Mean±SD	5.91±4.1	6.38±4.3	
**Marriage duration(years)**			
<5	14(35)	15(37.5)	0.763 ^**^
5–10	19(47.5)	16(40)	
>10	7(17.5)	9(22.5)	
Total	40(100)	40(100)	
Mean±SD	6.6±3.6	6.7±3.8	

* Independent sample t-test,

** Chi- square test,

*** Fisher exact test

To measure the changes in the total scores of infertility stress before, immediately after, and one month after the intervention, repeated measures test was used. Repeated measures test indicated that length of time had affected the total scores of infertility stress (p<0.001) and there was a significant difference between the two groups (p<0.001). The results of the study also showed the effectiveness of counseling on infertility stress (Table 2).

**Table 2.** Trend of infertility stress score i before, after and one month after counselling

**Factor**	**Group**	**Before (M±SD)**	**After (M±SD)**	**One month after (M±SD)**	**Mauchly test**	**Repeated**	**Measure**

						**Within group**	**Between group**
**Social concern**							
	Intervention	30.57±7.62	27.82 ±6.45	26.77±6.42	p<0.001	F=37.503 p<0.001	F=32.786 p<0.001
	Control	33.95±6/41	33.85±6.38	33.82±6.12			
**Sexual concern**							
	Intervention	26.30±8.93	24.10±7.86	22.87±7.52	p<0.001	F=33.496 p<0.001	F=39.514 p<0.001
	Control	25.97±6.71	26.02±6.75	26.12±6.72			
**Relationship concern**							
	Intervention	30.42±9.27	29.12±8.92	27.70±8.37	p=0.003	F=25.223 p<0.001	F=28.173 p<0.001
	Control	31.85±4.76	31.87±4.71	31.92±4.77			
**Rejection of childless lifestyle**							
	Intervention	29.45±4.79	21.97±4.55	20.32±4.36	p=0.003	F=114.017 p<0.001	F=125.323 p<0.001
	Control	29.30±4.78	29.27±4.76	29.62±4.47			
**Need for parenthood**							
	Intervention	41.97±6.51	40.55±5.17	37.32±5.12	p<0.001	F=52.132 p<0.001	F=57.602 p<0.001
	Control	43.12±5.16	43.20±5.16	43.25±5.15			
**Total stress score**							
	Intervention	158.72±30.83	143.57±26.49	135.00±24.73	p<0.001	F=137.593 p<0.001	F=149.473 p<0.001
	Control	164.20±21.37	164.22±21.19	164.75±20.60			

The results of repeated measures indicated that length of time had affected the general marital gender roles (p=0.001) and sexual and reproductive gender roles (p=0.001). Furthermore, there was a significant difference between both study groups in each domain (p=0.001) (Table 3).

**Table 3.** Trend of the gender role score before, after and one month after counselling

**Factor**	**Group**	**Before (M±SD)**	**After (M±SD)**	**One month after (M±SD)**	**Mauchlytest**	**Repeated**	**Measure**

						**Within group**	**Between group**
**Marital roles in sexual and reproductive health**							
	Intervention	37.47±3.51	39.30±2.73	40.60±2.69	p=0.001	F=32.779 p=0.001	F=32.896 p=0.001
	Control	38.35±3.21	3.19±38.32	3.19±38.35			
**Marital public roles**							
	Intervention	54.70±3.08	55.37±2.66	55.45±3.10	p=0.030	F=17.748 p=0.001	F=10.192 p=0.001
	Control	54.67±4.15	54.62±4.34	57.45±5.07			
	Control	93.02±4.87	92.95±5.19	95.80±5.70			

## Discussion

Results of the study demonstrated the effect of the counselling on total scores of infertility stress and all its domains. It should be noted that infertility is considered as a psychosocial crisis and counseling can be regarded as an integral part of the multi-faceted approach to infertility treatment; thus, counselling for infertile couples currently needs expertise and certain qualities (17).

Results of a systematic review study on 3401 infertile women who had received psychosocial interventions such as cognitive-behavioral counseling, infertility disclosure, mental awareness, over one week to six months indicated a reduction in stress and an increased chance of becoming pregnant (18).

Our results showed effects of counseling on social concerns in infertile women. Many studies have shown the effects of counselling on strengthening social relationship in infertile individuals with their surrounding environment which has been described as a coping strategy (27). Fear of stigmatization in infertile couples can often reinforce behaviors beyond control (28) and intensify social isolation (29). Some similar studies showed impact of psychological counselling on social relationships, quality of life, coping strategy with infertility, childless living, and marital relationships in couples (30). As this marital relationship is formed correctly, it helps share their feelings for having a child or frustration with having no children (31).

Our findings revealed the impact of counselling on sexual concerns. It should be noted that due to limitation of sexual relationships to ovulation and fertility days in the menstrual cycle during infertility, sexual relationships lose the enjoy of unpredictability and are merely concentrated on reproduction. So, sexual relationships are out of pleasure and romance relationship and target only pregnancy (32). This scheduled intercourse can consequently disrupt sexual life in the long term. Sexual relationships without any pregnancy can even bring about a sense of failure in a person and affect their body image (33). The results of the studies had indicated the effects of counselling on improving sexual satisfaction and reducing sexual concerns in infertile couples (34, 35).

The findings of this study showed effects of group counselling on the need for parenthood and childless living. In this regard, Rabiepour et al. reported that collaborative approaches training had influenced the need for parenthood in infertile women (23). There are results showing that infertile women compared to male are less likely to be satisfied and they could not also imagine a childless lifestyle. Such women also keep away from children and pregnant women and search for more information to treat infertility with a tendency to continue treatments despite low success rates (36).

The results of this study revealed the effect of counselling on gender-role attitudes in domain of sexual and reproductive issues. Our results showed 92.5% of women had an intermediate gender role attitude and 7.5% of them had traditional attitudes. Traditional attitude of female role included nurturing, motherhood, dependence, passivity, tolerance, emotions, as well as devotion (37). Some of studies showed the relationship between gender-role attitudes and infertility stress (12). Traditional gender-role attitudes were also significantly correlated with higher rates of depression (14). Women with traditional gender-role attitudes were putting up more pressure in the role of being a mother and also tolerated more social stigma during infertility (38). Study by Greiland et al. revealed that women with a male gender-role attitude such as independence and self-confidence could suffer from less stress (39).

In infertility counselling, risk level in a couple should be considered; for example, in female infertility, special psychological interventions for women due to social pressures and treatment processes should be designed (27).

In this study, there were attempts to provide counselling on causes, treatment methods and side effects, relaxation techniques, sexual and reproductive rights, gender-role attitudes, communication skills and sexual relationship with spouse in order to moderate levels of stress.

There were minimal start-up costs associated with the implementation of these counselling group and pre-exiting equipment was used and counselling sessions were held in hospital conference meeting. Counselling group could reduce stress and improve emotional needs of infertile women. The desire for participating in these counselling groups has been realized. Many women expressed emotional, physical, and psychological reactions towards infertility. They stated counselling groups have been very helpful who helped us to discuss our struggles with others in the same boat.

With regard to low cost of implementation, it was suggested that all infertile women participate in counselling sessions.

Limitations of this research included lack of possibility of holding group counseling sessions with husbands, due to unwillingness of them for participating in sessions and cultural barriers; therefore, it was recommended to conduct a study using a counselling program designed for couples. Another limitation was the inability to control use of other sources of information. One other constraint of this study was the duration of counseling process, which was measured only one month after the intervention. This study has also limited generalizability, as the sample was women with female infertility factor; sampling place was confined to one treatment center, which was the only center available at the time of sampling.

## Conclusion

Group counselling designed based on the guideline by the BICA was effective on decreasing infertility stress in women in dimensions of the social concerns, sexual concerns, relationship, childless living, need for parenthood and gender role attitude. It is recommended that group counselling for all infertile women be established and more studies are needed to investigate the effect of group counselling on stress and gender role of infertile couples, and impact of group counselling on outcome of infertility treatment.
